# Associations between knee pain and knee-loading physical activities at work and leisure – a cross-sectional study based on accelerometer measurements

**DOI:** 10.1186/s12891-025-08589-w

**Published:** 2025-04-08

**Authors:** Margareta Törnblom, Emma Haglund, Ann Bremander, Anna Nilsdotter, Maria LE Andersson, Pasan Hettiarachchi, Peter J Johansson, Magnus Svartengren, Katarina Aili

**Affiliations:** 1 Department of Clinical Sciences, Section of Rheumatology, Lund University, Lund, Sweden; 2 Spenshult Research and Development Centre, Halmstad, Sweden; 3 Department of Rehabilitation, Helsingborg Hospital, Helsingborg, Sweden; 4 Department of Environmental and Biosciences, School of Business, Innovation and Sustainability, Halmstad University, Halmstad, Sweden; 5 Danish Hospital for Rheumatic Diseases, University Hospital of Southern Denmark, Sønderborg, Denmark; 6 Department of Orthopaedics, Institute of Clinical Sciences, Sahlgrenska Academy, Gothenburg University, Gothenburg, Sweden; 7 Department of Orthopaedics, Sahlgrenska University Hospital, Gothenburg, Sweden; 8 Department of Medical Sciences, Occupational and Environmental Medicine, Uppsala University, Uppsala, Sweden; 9 Occupational and Environmental Medicine, Uppsala University Hospital, Uppsala, Sweden; 10 Department of Health and Sport, School of Health and Welfare, Halmstad University, Halmstad, Sweden

**Keywords:** Knee osteoarthritis, Objective measurement, Knee load, Physical activity, Physical effort at work

## Abstract

**Background:**

Knee pain is often an early sign of knee osteoarthritis (KOA). Physical activities (PA) constitute the recommended regime to those affected. However, knee-loading PA at work is linked to an increased risk for KOA. The primary aim of this study was to investigate associations between knee pain and accelerometer-measured knee-loading PA, at work and leisure respectively. The secondary aim was to investigate knee-related problems in relation to self-reported physical effort at work.

**Methods:**

This cross-sectional study included 107 working participants (aged 30–67) with knee pain. Knee pain was evaluated using the Knee Injury and Osteoarthritis Outcome Scale (KOOS), subscale Pain. Knee-loading PA (including daily steps, time in upright position, stair walking), and sitting/lying were measured by accelerometer for one week. Each knee-loading PA was analysed separately for the measurement periods: (1) total period, (2) time at work, and (3) leisure on workdays. Knee-related problems were evaluated by the KOOS subscales Symptoms, Activities of Daily Living, Function in Sport and Recreation, and Quality of Life. Analyses were made with linear regression, and stratified by high or low self-reported physical effort at work.

**Results:**

Participants with more knee pain walked on average fewer steps per day, and spent less time in an upright position during leisure on workdays, unstandardized coefficient (β) = 0.001, *p* = 0.044, β = 0.075, *p* = 0.001 respectively, i.e. spent less time in knee-loading PA. The associations were stronger for those reporting high physical effort at work, β = 0.116, *p* = 0.016. Participants with high physical effort at work rated their (knee-related) quality of life worse. There were no associations between knee pain and knee-loading PA during work hours.

**Conclusions:**

Participants with more knee pain were less physically active during leisure, with stronger associations among those with higher physical effort at work. Those reporting high physical effort at work had worse (knee-related) quality of life compared to participants reporting low effort at work. This highlights the importance of taking knee-loading PA at work and leisure into account when recommending exercise regimes to individuals with knee pain.

**Trial registration:**

ClinicalTrials.Gov (NCT04928170), Date of registration: 2017-12-20.

**Supplementary Information:**

The online version contains supplementary material available at 10.1186/s12891-025-08589-w.

## Background

Knee pain is a common symptom among the adult population. Over time, 86% of the population with knee pain develop radiographic knee osteoarthritis (KOA) [1]. KOA affects the joint and surrounding tissues, causing symptoms, such as pain, stiffness, and signs of crepitus, joint swelling, and bony enlargements. It might lead to joint failure and structural damage [2]. KOA is characterised by functional impairment, activity limitations and participation restrictions, which, in the long run, have consequences for both individuals and society [3]. In addition, frequent knee pain and more knee-related problems are associated with an increased risk of developing chronic widespread pain [4, 5]. Age, obesity, female sex, work with knee-loading physical activities (PA), previous knee injury, and impaired physical function are independently considered to increase the risk of developing KOA [2].

Early preventive actions are likely to reduce the modifiable risk factors linked to the development of KOA, and contribute to decreasing progression rate in the long run [6, 7]. Education, exercise, and, if needed, weight loss have shown a positive effect on pain and physical function and are the recommended first-line treatments for individuals with KOA [8]. In terms of exercise, this includes being physically active at a level in line with World Health Organization (WHO) recommendations [9]. This means that individuals, aged 18–64, with chronic conditions or disabilities, e.g., KOA, are recommended to be engaged in at least 150 min of moderate PA per week, or 75 min of vigorous PA per week [9]. However, less than one fifth with KOA reach 10,000 steps per day [10], and lower levels of PA lead to an increased risk of comorbidity [11]. The PA recommendations for individuals with KOA do not consider whether the PA is performed at work or leisure, which may be of relevance. It is also unclear whether the PA recommendations need to be adapted to the individual’s physical effort at work.

While international recommendations promote exercise as a first-line treatment for KOA [8], and for general health improvement [9], several studies have highlighted a contrasting concern. Occupations involving high physical effort may negatively impact health, increasing the risk of cardiovascular disease and mortality [12, 13]. This anomaly is described as the “PA paradox”. The PA paradox can be explained by the fact that occupational PA often limits a worker’s ability to choose the frequency, intensity and type of PA, and the posture, thereby increasing the risk of negative health effects. Similarly, PA may negatively impact health if the work tasks are prolonged, repetitive, or static, or if there is insufficient opportunity for recovery. This seems to be in contrast to PA at leisure [14]. A similar PA paradox may apply to individuals with KOA. While PA in general seems beneficial to those with KOA, the risk for developing the disease is higher among individuals working in occupations with knee-loading PA, e.g., heavy lifting/carrying, kneeling/squatting, climbing ladders/stairs, and/or prolonged standing and walking [15, 16]. In contrast to this, sitting at work seems to be protective against KOA [16]. In this study, we have used accelerometers to objectively measure knee-loading PA in order to minimise potential inaccuracies e.g. response bias, related with self-reported measures [17]. Estimates from self-reported PA tend to be higher compared to objectively measured PA. In addition, correlations between objectively measured PA and self-reported PA in individuals with KOA have been reported weak [18]. To our knowledge, few studies have assessed knee-loading PA in individuals with knee pain, and separated between PA performed during work and leisure.

Previous research indicates links between musculoskeletal pain, high physical effort at work, and spending less time in PA at leisure [19, 20]. General practitioners/health care professionals should consider that there might be differences between PA at work and PA at leisure when caring for individuals with knee pain, as a possible symptom of KOA. More knowledge on the connections between knee-loading PA and pain is needed, to improve the personalized recommendation of PA in individuals with knee pain.

## Methods

### Aim

The primary aim of this study was to investigate associations between knee pain and accelerometer-measured knee-loading PA at work and leisure, respectively. The secondary aim was to investigate knee-related problems in relation to self-reported physical effort at work.

### Study design

This cross-sectional study was conducted in southern Sweden, and includes baseline data from a sub-sample of the Halland osteoarthritis cohort (HALLOA), an ongoing longitudinal study [21] (ClinicalTrials.Gov NCT04928170). All eligible participants were examined by a general practitioner before inclusion. The general practitioner took a medical history, enquiring about previous knee injuries and characteristic symptoms of inflammatory rheumatic diseases. A thorough examination of the knee joints was performed, including inspection, palpation, and stability testing. In addition, rheumatoid arthritis was ruled out by a blood sample test for anticyclic citrullinated peptide [21].

### Participants and recruitment

All participants from HALLOA who were working were approached for inclusion in this study. After receiving information about the study, 118 individuals, aged 30–67, agreed to participate. Those included had knee pain and no former known radiographic KOA, cruciate ligament injury or inflammatory rheumatic disease. Data were collected between November 2018 and December 2019. All participants gave their written and informed consent prior to participation. This study was approved, in accordance with the Declaration of Helsinki, by the Regional Ethical Review Board in Lund, Sweden approved the study (nos. 2016/229, 2017/253, 2018/602).

### Assessment of knee pain

The primary outcome, knee pain, was self-reported with the subscale Pain, in the Knee Injury and Osteoarthritis Outcome Score (KOOS) [22, 23]. KOOS has shown good validity, reliability, and responsiveness in individuals with knee injuries and KOA. KOOS Pain contains nine items, starting with: “How often do you experience knee pain?”. The following eight questions ask: “What amount of knee pain have you experienced the last week during the following activities?”. The activities are as follows: Twisting/pivoting on your knee, straightening knee fully, bending knee fully, walking on flat surface, going up or down stairs, at night while in bed, sitting or lying, and standing upright. Each item is answered on a five-point Likert scale, ranging from 0 (no problems) to 4 (extreme problems), calculated to a score of 0-100 (worst to best).

### Assessment of knee-loading physical activities

#### Accelerometery

Each participant was equipped with a single tri-axial accelerometer device (Axivity AX3, Axivity Ltd., Newcastle upon Tyne, UK), positioned at the right thigh (anterior, approximately midway between the iliac crest and patella) with skin-friendly tape. The participants were asked to wear the accelerometer at all times during seven consecutive days and nights, and were encouraged to proceed with their everyday activities as usual, with the exception of taking a bath or swimming. A seven-day measurement period is routine when evaluating PA [24]. During the week-long accelerometer-measurement, the participants also sent daily reports on work time and sleep time via short message service (SMS). This information was used to identify time periods of work, leisure and sleep during the processing of accelerometer data.

#### Data processing

The accelerometer was initialized for recording and data were downloaded with the software (Open Movement GUI, version 1.0.0.30). The acceleration was sampled in a dynamic range of ± 8 G_n_ in three directions, where 1 G_n_ is comparable to the gravity of the Earth. The sampling frequency was recorded at 25 Hz. The data were processed using the validated Acti4 Algorithm [25–27], incorporated in the ActiPASS software, ActiPASS [28, 29]. ActiPASS calibrates the raw accelerometer data first using an auto-calibration algorithm [30]. Afterwards, an individual-specific calibration is carried out by ActiPASS to account for the minor deviations in the placement of the accelerometer and individuals’ upright posture by its automatic reference-position algorithm. ActiPASS then utilizes above device and individual specific calibrated raw accelerometer data to identify movements and the inclination of the thigh relative to the line of gravity. This information is used to classify physical behaviours, including sitting, lying, standing, moving (periods in a standing posture with certain movements, such as intermittent steps without purposeful walking), walking, running, bicycling, stair walking. Additionally, ActiPASS calculates the total number of steps taken from all activities combined, including walking, running, and stair walking. Unlike many other step-counting tools, ActiPASS does not identify steps during moving (short intermittent stepping without purposeful walking) activities. Non-wear periods were automatically detected by the ActiPASS software. Wear time less than 20 h per day was not included as a valid day. Time spent in bed, according to the participants’ daily report, was excluded from the analysis.

#### Definition of knee-loading physical activities

**Fig. 1:**
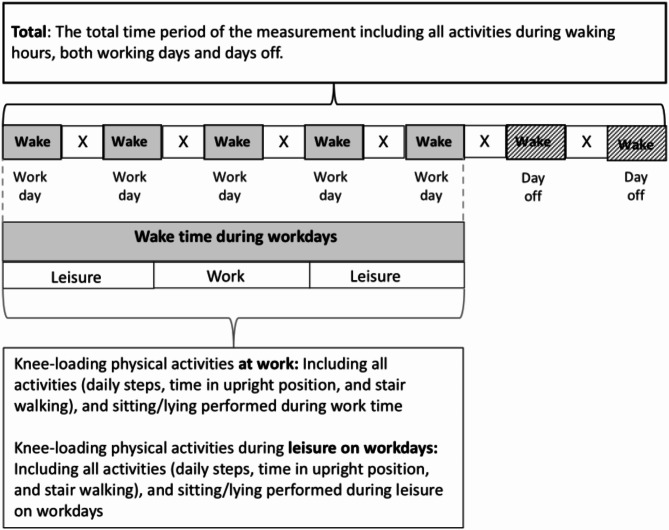
Illustrating data used from a 7-day accelerometer measurement when presenting “total” activity, and when presenting data on activities split by work and leisure during workdays. Data on activities from sleep time (time in bed, marked as X in the figure) were excluded

In this study, knee-loading PA were defined as: (1) average number of daily steps (including walking, stair walking, and running), (2) average time (minutes) per day spent in upright position ((including standing, moving (intermittent steps but without purposeful walking), walking, running and stair walking)), and (3) average time (minutes) per day spent in stair walking (Fig. 1). The knee-loading PA, along with time spent sitting/lying, were analysed separately for different measurement periods, describing respective activity performed during: (1) total time period of the measurement (labelled as “Total”), including all activities during waking hours, both working days and days off, (2) time at work, and (3) leisure on workdays, including the time spent in leisure on workdays (excluding days off) (Fig. 1).

### Assessment of knee-related problems

To investigate the distribution of knee-related problems, additional data were collected by the four remaining KOOS subscales each calculated to a score of 0-100 (worst to best) [22, 23]: Symptoms, Activities in daily living (ADL), Function in sport and recreation (Sport/Rec), and Quality of life (QoL). These subscales contained varying numbers of items; Symptoms (7 items), Activities in daily living (17 items), Function in sport and recreation (5 items), and knee-related Quality of Life (4 items).

### Assessment of self-reported physical effort at work

Participants were asked to report how physically demanding their current work was [31, 32]. The physical effort at work was graded on a semi-continuous scale about perceived exertion according to Borg [33]. The scale ranges from 6 to 20, and includes explanatory anchors: 7 equals “*very*,* very light”*, 9 equals “*very* light”, 11 equals “*fairly light*”, 13 equals “*somewhat hard*”, 15 equals “*hard*”, 17 equals “*very hard*”, and 19 equals *“very*,* very hard”*. Physical effort was categorized into “high” or “low” group. A statistical cut-off value was set based on the median 12, high effort included 13–20 (somewhat hard or more), and low effort included 6–12 (below somewhat hard).

### Assessment of potential confounders

Obesity and weakness of the quadriceps are considered risk factors for developing KOA [2]. Having chronic widespread pain is more common in individuals with knee pain than in the general population [4]. Body mass index (BMI) (kg/m^2^), quadriceps strength, and number of pain sites was assessed as potential confounders. Body weight (kg) and height (m) were measured, and BMI (kg/m^2^) was calculated. The maximal voluntary isometric contraction (MVIC) of the quadriceps muscle was assessed in an upright sitting position, with 90° knee and hip flexion. Measurement was performed with a hand-held dynamometer against the tibia (Commander Muscle Tester, 2016 JTECH Medical MN084_D. JTECH Medical Industries, Midvale, USA) [34, 35]. The MVIC was repeated three times per side, and the mean peak value of both sides was calculated and registered in newtons (N). Localisation and number of painful sites of pain lasting more than three months were assessed by a pain mannequin [36]. The pain mannequin has 18 predefined body regions in the musculoskeletal system.

### Assessment of variables included for descriptive purposes

The participants reported their current occupation. Their occupations were then categorised based on likely exposure to knee-loading PA at work, as: (1) *unexposed* (e.g., medical secretary, HR administrator and student), (2) *partially exposed* (e.g., teacher, production manager, and engineer), (3) *exposed* (e.g., nursing staff, preschool teacher, construction worker, farmer, service and retail workers). In this study, an exposed work means an exposure of the lower extremity, and includes prolonged standing, walking, squatting, or heavy lifting, which is in line with exposures used when developing lower body job exposure matrix [37]. The categorisation was made based on clinical experiences after consensus was achieved within the research group, which included physiotherapists, ergonomists and a physician specialized in occupational medicine.

Self-reported PA, the participants estimated how much of their weekly leisure time they spent in moderate PA (such as brisk walks, light cycling or gardening), or in vigorous PA (e.g., running and ball sports). Total amount of weekly PA (minutes/week) was calculated ((vigorous PA *2) + moderate PA) [38], and categorized into three groups: <90(minutes/week, 90–150 min/week, and > 150 min/week).

### Data analysis

The data was normally distributed and analysed using parametric tests. Frequency, mean and standard deviation (SD) were used when describing the data and, for group comparisons, the Chi-square test or independent samples T-test were used. For the accelerometer measurements, the mean was the mean of means, i.e. first a weekly mean of the knee-loading PA for each participant was calculated, and then a weekly mean for the cohort was calculated. Associations between knee pain (KOOS Pain) and knee-loading activities, and sitting/lying, during: (1) the total measurement period, (2) time at work, and (3) leisure on workdays, and covariates were tested with univariate linear regression analysis. Multivariate linear regression analyses were then performed for each of the knee-loading PA separately. Only potential confounders that were significantly associated with KOOS Pain in the univariate linear regression analysis were included as confounders in the multivariate analysis. Additional subgroup analyses were performed after stratification by self-reported (high/low) physical effort at work. A sensitivity analysis were made due to extreme values from one participant (who walked on average > 38,000 steps per day). All analyses were performed in IBM SPSS Statistics for Windows, Version 27.0 (Armonk, USA).

## Results

In all 118 participants were eligible for inclusion. The sensitivity analysis showed that inclusion of the outlier changed the results of the regression analysis (for detailed results of the analysis, see Supplementary material 1). A decision was thus made to exclude this extreme value from the analysis, leaving data from 117 participants for inclusion in the study. Of the remaining 117 participants, 10 were excluded from further analysis, due to missing data on KOOS Pain (*n* = 1), errors during accelerometer measurement (*n* = 3), missing reports of work hours (*n* = 2), being sick or off work during the measurement (*n* = 4). Of those excluded, 70% were women, had a mean age of 50 years (SD 10), and BMI (kg/m^2^) of 26.9 (SD 3.8). This did not differ from those included in the analysis.

The final analyses were based on 107 participants, of which 68% (*n* = 73) were women. The mean age was 52 years (SD 8), BMI (kg/m^2^) 26.0 (SD 4.5), and KOOS Pain 73.4 (SD 16.8) (Table 1). Three out of four participants worked in the daytime; the rest reported working nights, shiftwork, or irregular schedules. The instruction was to wear the accelerometer for one week. In total, the participants wore the accelerometer for between three and nine days, with a mean of 6.3 days (SD 1.1), of which they spent time at work during one to eight days, mean of 3.8 days (SD 1.2). During the measurement, participants walked on average approximately 11,400 steps per day, and of these, more than 6,000 steps were taken during working hours (Table 1).

**Table 1:** Characteristics of the study participants, *n* = 107

	Mean (SD)
Age, (years)	52.3 (8.2)
Sex (women), n (%)	73 (68,2)
Body Mass Index, BMI (kg/m^2^)	26.0 (4.5)
KOOS Pain	73.4 (16.8)
KOOS Symptoms	55.6 (12.8)
KOOS ADL	80.1 (15.3)
KOOS Sport/Rec	47.3 (26.7)
KOOS QoL	47.0 (17.0)
MVIC of quadriceps, (Newton), *n* = 97	277.9 (106.9)
Number of pain sites, (0–18), *n* = 105	3.2 (3.8)
Physical effort at work, *n* = 101	12 (2.9)
*Exposure to knee-loading PA at work*	
Unexposed, n (%)	37 (34.6)
Partially exposed, n (%)	10 (9,3)
Exposed, n (%)	60 (56.1 )
*Physical activities at leisure*, *n* = 106	
<90 (minutes/week), n (%)	10 (9.4)
90–150 (minutes/week), n (%)	14 (13.2)
>150 (minutes/week), n (%)	82 (77.4)
**Accelerometer measurements**	
*Daily steps*	
Total^†^	11,399 (3757)
At work	6292 (3501)
Leisure on workdays	5015 (2305)
*Upright position*,* (minutes/day)*	
Total^†^	412 (103)
At work	237 (115)
Leisure on workdays	183 (64)
*Stair walking*,* (minutes/day)*	
Total^†^	8.3 (5.3)
At work	4.1 (5.0)
Leisure on workdays	4.1 (3.2)
*Sitting/lying*,* (minutes/day)*	
Total^†^	542 (95)
At work	251 (102)
Leisure on workdays	288 (84)

**KOOS =** Knee injury and Osteoarthritis Outcome Score (0-100 worst-best). **ADL** = Function, daily living, **Sport/Rec** = Function, sports and recreational activities. **QoL** = Quality of Life. **MVIC** = Maximal voluntary isometric contraction. **Physical effort at work** = (6–20 scale, no effort - maximum effort). **Daily steps** = average daily steps. **Upright position =** including standing, moving, walking, running and stair walking. **Sitting/lying** = (when awake) in sitting or lying position. ^†^ Including all activities during waking hours, both working days and days off

### Associations between knee pain and knee-loading physical activities at work and leisure

Positive associations were found between KOOS Pain and knee-loading PA; for daily steps and time spent in an upright position during the total measurement period, and during leisure on workdays. This indicates that more knee pain, according to KOOS Pain, was associated with spending less time in knee-loading PA (at least during leisure time). No associations were found between knee pain and knee-loading PA at work. Additionally, no associations were found for the variables stair walking and sitting/lying, regardless of whether the activities were specified as being performed during the total measurement period, at work, or leisure on workdays (Table 2). Adjusted for BMI and number of pain sites, the multivariate regression analysis showed comparable results to the univariate analysis, except for the association between knee pain and daily steps for the total measurement period, which became non-significant (*p* > 0.05) (Table 2).

**Table 2:** Linear regression analysis and associations with knee injury osteoarthritis outcome score, subscale pain

		Univariate		Adjusted for BMI and number of pain sites	
	*n*	B (95% CI)	*p*-value	B (95% CI)	*p*-value
Age, (years)	107	-0.218 (-0.612; 0.175)	0.274		
*Sex*	107	-5.526 (-12.376;1.324)	0.113		
Body Mass Index (BMI), *(kg/m*^*2*^*)*	107	-0.831 (-1.539; -0.123)	**0.022**		
MVIC of quadriceps (Newton)	97	-0.006 (-0.038;0.026)	0.693		
Number of pain sites, (0–18)	105	-1.416 (-2.201; -0.632)	**0.001**		
**Accelerometer measurements**					
*Daily steps*					
Total^†^	107	0.001 (0.000;0.002)	**0.019**	0.001 (-0.000;0.001)	0.134
At work	107	0.000 (-0.001;0.001)	0.521	0.000 (-0.001;0.001)	0.883
Leisure on workdays	107	0.002 (0.001;0.003)	**0.007**	0.001 (0.000;0.003)	**0.044**
*Upright position (minutes/day)*					
Total^†^	107	0.042 (0.012;0.073)	**0.007**	0.034 (0.005;0.062)	**0.023**
At work	107	0.006 (-0.023;0.034)	0.697	0.002 (-0.023;0.028)	0.867
Leisure on workdays	107	0.091 (0.044;0.138)	**< 0.001**	0.075 (0.030;0.120)	**0.001**
*Stair walking*,* (minutes/day)*					
Total^†^	107	0.298 (-0.315;0.912)	0.337	0.083 (-0.486;0.653)	0.772
At work	107	-0.030 (-0.678;0.617)	0.926	-0.197 (-0.783;0.389)	0.507
Leisure on workdays	107	0.934 (0.065;1.933)	0.067	0.788 (-0.120;1.695)	0.088
*Sitting/lying (minutes/day)*					
Total^†^	107	-0.030 (-0.064;0.004)	0.082	-0.023 (-0.054;0.009)	0.159
At work	107	0.002 (-0.029;0.034)	0.891	0.002 (-0.027;0.031)	0.877
Leisure on workdays	107	-0.030 (-0.068;0.008)	0.123	-0.029 (-0.063;0.006)	0.099

**B** = unstandardized coefficient. **KOOS =** Knee injury and Osteoarthritis Outcome Score (0-100 worst-best). **MVIC** = Maximal Voluntary Isometric Contraction. **Daily steps** = average daily steps. **Upright position =** including standing, moving, walking, running and stair walking. **Sitting/lying** = (when awake) in sitting or lying position. ^†^Including all activities during waking hours, both working days and days off. P-value in bold typeface ≤ 0.005

### Knee-related problems in relation to self-reported physical effort at work

**Fig. 2:**
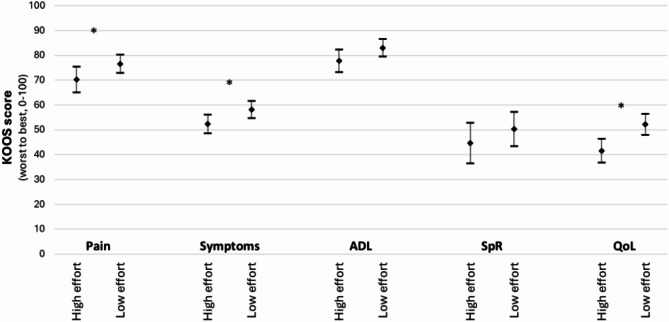
Comparisons of Knee Injury and Osteoarthritis Outcome Score (KOOS) subscales categorised by participants reporting high or low self-reported physical effort at work. Presented as mean and 95% confidence interval. ADL = KOOS Activities in Daily living, SpR = KOOS Sport/Recreation, and QoL = KOOS knee-related Quality of Life. *p-value < 0.005

In this study, 48 participants (45%) reported high physical effort at work. These participants scored lower (i.e. worse) on KOOS Pain, mean 70.3 vs. 76.6, *p* = 0.050, KOOS Symptoms 52.4 vs. 58.2, *p* = 0.022, and KOOS QoL, 41.6 vs. 52.2, *p* = 0.001, compared to those with low physical effort at work. The difference between high and low physical effort at work, for KOOS ADL and KOOS Sport/Rec was not statistically significant, mean 77.8 vs. 83.1, *p* = 0.066, and mean 44.7 vs. 50.3, *p* = 0.284, respectively (Fig. 2).

### Associations between knee pain, knee-loading physical activities at work and leisure, stratified by self-reported physical effort at work

Among individuals whose occupations were classified as exposed to knee-loading PA at work, 88% reported high physical effort. In contrast, only 6% of those in partly exposed occupations and 6% of those in unexposed occupations reported similar levels of physical effort (Table 3).

According to the accelerometer measurement of the knee-loading PA, the group who reported high physical effort at work walked on average more daily steps at work, 7506 vs. 5327, *p* = 0.001. Furthermore, they spent more time (minutes) in an upright position during both the total time period of the measurement, 442 vs. 387 *p* = 0.007, and at work, 287 vs. 195, *p* < 0.001, compared to those rating low physical effort. Furthermore, participants reporting high physical effort at work spent on average slightly more time (minutes) in stair walking at work, 5.3 vs. 3.2, *p* = 0.044, and less time (minutes) in sitting/lying, both during the total measurement period, 499 vs. 578, *p* < 0.001, and at work, 195 vs. 294, *p* < 0.001, compared those reporting less physical effort at work (Table 3).

**Table 3:** Description of objectively measured physical activities in participants reporting high/low physically effort at work

	High physical effort at work Mean (SD) (*n* = 48)	Low physical effort at work Mean (SD) (*n* = 55)	*p*-value
Age, (years)	53.6 (7.9)	51.5 (8.2)	0.181
Sex (female), n (%)	33 (68.8)	38 (69.1)	0.970
BMI, *(kg/m*^*2*^*)*	26.5 (4.2)	25.8 (4.7)	0.410
Number of pain sites, (0–18)	4.0 (4.0)	2.6 (3.5)	0.062
*Knee-loading exposure at work*, n (%)			**< 0.001**
Unexposed	3 (6.3)	33 (60.0)	
Partially exposed	3 (6.3)	7 (12.7)	
Exposed	42 (87.5)	15 (27.3)	
**Accelerometer measurements**			
*Daily steps*			
Total^†^	12 151 (4159)	10 829 (3346)	0.077
At work	7506 (3392)	5327 (3362)	**0.001**
Leisure on workdays	4802 (1958)	5307 (2567)	0.270
*Upright position*,* (minutes/day)*			
Total^†^	442 (102)	387 (100)	**0.007**
At work	287 (110)	195 (103)	**< 0.001**
Leisure on workdays	179 (52)	189 (72)	0.440
*Stair walking*,* (minutes/day)*			
Total^†^	9.0 (6.7)	7.8 (3.8)	0.304
At work	5.3 (6.4)	3.2 (3.3)	**0.044**
Leisure on workdays	3.9 (2.8)	4.5 (3.5)	0.345
*Sitting/lying*,* (minutes/day)*			
Total^†^	499 (93)	578 (83)	**< 0.001**
At work	195 (79)	294 (99)	**< 0.001**
Leisure on workdays	282 (83)	294 (87)	0.477

Analysed with Chi-square test or Independent samples T test. **Physical effort at work** = self-reported (6–20 scale, no effort - maximum effort) dichotomized into two groups: high (≥ 13) or low (< 13). **BMI =** Body Mass Index (kg/m^2^). **Knee-loading exposure** = based on current occupation. **Daily steps** = average daily steps. **Upright position =** including standing, moving, walking, running and stair walking. **Sitting/lying** = (when awake) in sitting or lying position. ^†^Including all activities during waking hours, both working days and days off. P-value in bold typeface ≤ 0.005

Within the group with high physical effort at work, positive associations were seen between KOOS Pain and time spent in an upright position during the total measurement B 0.055 (95% CI 0.006 to 0.104; *p* = 0.028), and during leisure on workdays 0.110 (95% CI 0.014 to 0.206; *p* = 0.025). The results indicate that more pain was associated with less time spent in an upright position (at least during leisure). Further, negative associations were seen between KOOS Pain and time spent in sitting/lying during the total measurement period − 0.056 (95% CI -0.110 to -0.002; *p* = 0.042) and during leisure on working days − 0.074 (95% CI -0.133 to -0.014; *p* = 0.016). The results indicate that more pain was associated with more time spent in sitting/lying position (at least during leisure). The associations seen were attenuated when adjusted for BMI and number of pain sites, and only the association with time spent in upright position during the total measurement period remained statistically significant 0.116 (95% CI 0.023 to 0.210; *p* = 0.016) (Table 4).

Within the group with low physical effort at work, positive associations were seen between KOOS Pain and daily steps during the total measurement period 0.001 (95% CI 0.000 to 0.002; *p* = 0.037). Positive associations were also seen between KOOS Pain and time spent in upright position during the total measurement period 0.049 (95% CI 0.014 to 0.084; *p* = 0.008), and during leisure on workdays 0.066 (95% CI 0.016 to 0.115; *p* = 0.010). The results imply that more pain is associated with fewer steps and less time spent in an upright position (at least during leisure time). When adjusting for BMI and number of pain sites, the associations between KOOS Pain and daily steps attenuated and were no longer significant. The other associations remained largely unchanged in the multivariate model (Table 4).

**Table 4:** Regression analysis, associations between knee-loading activities and KOOS (Pain), stratified by physical effort at work

	High physical effort at work (*n* = 48)				Low physical effort at work (*n* = 55)			
	**Univariate** B (95% CI)	P-value	**Multivariate** B (95% CI)	P-value	**Univariate** B (95% CI**)**	P-value	**Multivariate** B (95% CI)	P-value
*Daily steps*								
Total^†^	0.001 (-0.000;0.002)	0.140	0.001 (-0.001;0.002)	0.354	0.001 (0.000;0.002)	**0.037**	0.001 (-0.000;0.002)	0.071
At work	0.001 (-0.001;0.002)	0.371	0.000 (-0.002;0.002)	0.986	0.001 (-0.001;0.002)	0.317	0.001 (-0.001;0.002)	0.288
Leisure on workdays	0.002 (-0.000;0.005)	0.102	0.002 (-0.000;0.005)	0.103	0.001 (-0.000;0.003)	0.104	0.001 (-0.000;0.002)	0.186
*Upright position (minutes/day)*								
Total^†^	0.055 (0.006;0.104)	**0.028**	0.047 (-0.004;0.098)	0.071	0.049 (0.014;0.084)	**0.008**	0.044 (0.007;0.081)	**0.019**
At work	0.034 (-0.013;0.081)	0.153	0.014 (-0.034;0.063)	0.558	0.013 (-0.023;0.050)	0.468	0.012 (-0.024;0.048)	0.509
Leisure on workdays	0.110 (0.014;0.206)	**0.025**	0.116 (0.023;0.210)	**0.016**	0.066 (0.016;0.115)	**0.010**	0.061 (0.009;0.112)	**0.022**
*Stair walking*,* (minutes/day)*								
Total^†^	0.457 (-0.324;1.237)	0.245	0.247 (-0.550;1.004)	0.535	-0.127 (-1.122;0.868)	0.798	-0.163 (-1.164;0.838)	0.746
At work	0.322 (-0.492;1.136)	0.430	0.036 (-0.779;0.850)	0.930	-0.725 (-1.868;0.418)	0.209	-0.703 (-1.840;0.435)	0.221
Leisure on workdays	1.039 (-0.799;2.878)	0.261	1.277 (-0.561;3.115)	0.168	0.612 (-0.451;1.675)	0.253	0.602 (-0.450;1.653)	0.256
*Sitting/lying (minutes/day)*								
Total^†^	-0.056 (-0.110;-0.002)	**0.042**	-0.047 (-0.102;0.008)	0.094	-0.036 (-0.080;0.009)	0.116	-0.032 (-0.078;0.014)	0.165
At work	0.006 (-0.060;0.073)	0.846	-0.005 (-0.071;0.060)	0.873	-0.023 (-0.061;0.015)	0.227	-0.020 (-0.057;0.018)	0.299
Leisure on workdays	-0.074 (-0.133;-0.014)	**0.016**	-0.061(-0.124;0.003)	0.060	-0.007 (-0.050;0.037)	0.763	-0.013 (-0.057;0.031)	0.549

Linear regression analysis. **B** = unstandardized coefficient. The multivariate analysis was adjusted for body mass index (BMI) and number of pain sites. **KOOS** = Knee injury and Osteoarthritis Outcome Score (100-0 best-worst). **Number of pain sites** = from 0 to 18 sites. **Physical effort at work** = self-reported (6–20 scale, no effort - maximum effort) dichotomized into two groups: high (≥ 13) or low (< 13). **Daily steps** = average daily steps. **Upright position =** including standing, moving, walking, running and stair walking. **Sitting/lying** = (when awake) in sitting or lying position. ^†^Including all activities during waking hours, both working days and days off. P-value in bold typeface ≤ 0.005

## Discussion

In this cross-sectional study, we investigated associations between knee pain and knee-loading PA, separating time during work from time during leisure. The participants with more knee pain walked on average fewer steps per day and spent less time in an upright position during leisure on workdays, i.e. spent less time in knee-loading PA. However, we found no associations between knee pain and knee-loading PA during work hours. The associations were somewhat stronger for participants with high self-reported effort at work for the variable time spent in upright position during leisure on workdays. Participants who reported high physical effort at work also reported worse pain and more symptoms, and lower QoL in the KOOS subscales, than those with low physical effort.

In this study, having more knee pain was linked to fewer knee-loading activities during leisure on workdays. A one-point worsening of knee pain, according to KOOS, was associated with, on average, one thousand fewer steps during leisure on a workday. Previous findings [39–41], on PA and daily steps in individuals with KOA, or at risk of developing KOA, have shown mixed results regarding knee pain and PA. In one such study, the degree of knee pain had an impact on PA, where individuals with greater knee pain were less likely to engage in moderately intense levels of PA, compared to those with no knee pain. However, there were no associations between different levels of knee pain and engaging in light intense PA [40]. Other studies have shown that knee pain was not connected to daily steps [39, 41]. It may be difficult to compare our study with the studies mentioned above. In our study, the participants were in work. In addition, the previous studies have not made the same distinction between PA at work and at leisure time as in the current study we have done.

The participants who reported high physical effort at work walked more and spent more time in an upright position at work, compared to those reporting low physical effort. Interestingly, they seemed to compensate for their knee-loading activities at work by spending both less time in knee-loading PA and more time in a sitting/lying position at leisure. Comparable associations were not seen regarding spending time in sitting/lying among the participants reporting low physical effort at work. Similar patterns of compensation have previously been described for working individuals after total knee arthroplasty [42]. In the current study, compensating with less knee-loading PA during leisure could be a way for the participants to manage their knee pain. The group reporting high physical effort at work may have a low degree of job decision latitude [43], i.e. low degree of control and ability to influence their work tasks, or ability to determine work positions to minimise their knee pain. However, this is only hypothetical, as the current study did not investigate the participants’ degree of job decision latitude. For most employees, the opportunity to decide which knee-loading PA to perform was greater at leisure, compared to at work. More daily steps at work, compared to number of daily steps during leisure, were found to be associated with increased risk of long-term sickness absence, and could be linked to the PA paradox [44]. Related to the PA paradox, Merkus et al. conducted a two-year longitudinal study on workers in construction and healthcare [20], investigating whether PA during work and leisure was related to overall musculoskeletal pain and whether different intensities had an impact. Their study indicated higher overall pain levels for those who spent more time in PA at work than at leisure [20]. Similar to the Merkus et al. study, we found in the current study a more pronounced association between more knee pain and less knee-loading PA at leisure among those who reported higher physical effort at work. In addition, we found that having a job with high self-reported physical effort was associated with having more knee-related problems, according to KOOS, especially according to the QoL subscale. The link between knee pain, knee-loading PA at work and the risk of developing KOA need to be further studied, to elucidate the PA paradox. However, as this is a cross-sectional analysis, it is not possible to draw any causal conclusions. A longitudinal follow-up may provide more knowledge on the relationship between knee pain and knee-loading PA.

Using accelerometers to assess knee-loading PA is beneficial, given that they are more reliable and valid than self-reported PA [18]. By attaching the accelerometer to the thigh instead of the waist, we were able to better specify a range of movement patterns and distinguish certain movements, such as sitting from lying down, or level walking from stair walking [26]. In addition, the participants were instructed not to remove the accelerometer until the end of the measurement, making it possible to maintain the specific and calibrated position on the thigh for the accelerometer. In this, our study differs from studies using waist-worn accelerometers, where participants often remove and attach the device themselves on a daily basis, which might affect the accuracy of the measurement. Even if the measurement is regarded as objective, there may still be some risk that participants’ activity levels can be affected by being measured. Nevertheless, a previous study in adolescents showed that accelerometer measurement did not affect their daily PA patterns [45], and this might also be true for the adults in the current study.

The accelerometer data used in this study were not yet validated against the WHO recommendations for PA, and therefore we also added self-reported data on PA. Although the intensity of PA in our study could not be measured, the number of steps can be considered as high. Despite knee pain, more than half of the participants walked 10,000 steps per day, or more. In a previous study, an average of 7,000 steps per day were found to be comparable with 150 min/week of moderate to vigorous PA, i.e. equal to the WHO recommendation of PA [46]. The number of daily steps differ between our study and a previous review and meta-analysis [10]. According to the their analysis, less than one fifth reached 10,000 daily steps [10]. However, our study is not fully comparable to the studies included in the meta-analysis, given that 63% had severe radiographic KOA, a higher mean age (at working age or slightly older), and BMI, compared to those included in the current study [10].

Our current study has both strengths and weaknesses that should be mentioned. One strength is that this is, to our knowledge, the first study to investigate associations between knee pain and accelerometer-measured knee-loading PA at work and leisure. We aimed for a measurement period of one week, which is the time period routinely used when assessing PA with accelerometers [24]. In our study, the length of the measurement period varied between the participants, which may have affected the results. However, this variation seems to be in line with larger cohort studies [47]. The sample size of this study can be considered small, and a further limitation is the lack of a control group. Therefore, the results should be interpreted with some caution, in particular the results from the analyses stratified by self-reported effort at work. However, the included participants had a wide range of occupations, were a heterogeneous group, and this may be an advantage, as the results could be more generalizable. However, it is important to remember that those included in the study do not represent a general population because, in our selection process, only volunteer, working individuals with knee pain were included, i.e. there could be selection bias. Working individuals generally tend to have better health status than non-working individuals, also known as the “healthy worker effect” [48].

Although it is not possible to draw causal conclusions from a cross-sectional study such as this, the associations noted in this study may be of support in primary care when caring for individuals with knee pain, regarding their knee-loading PA at work and leisure. Even though the participants in this study were not primary care patients, knee pain is prevalent in the adult population and often develops to KOA [1]; therefore, they could be seen as potential patients for primary care. The total daily knee-loading PA are likely to be accumulated and it may be important to have a balance in terms of total time devoted to it during the day [49]. There is as yet no established recommendation on the exact type of PA, dose or what is the best modality for individuals with knee OA. But still, it is important to emphasise that exercise has a positive effect on pain and function [8]. Furthermore, it is essential that primary care professionals, such as physiotherapists, identify patients with knee pain who work in knee-loading occupations and assess their work situation [8], in order to advise them regarding their PA. If possible, this group of patients should be given personalized recommendations of PA that can be done in leisure time, but preferably without significantly negatively affecting their knees.

## Conclusion

Participants reporting more knee pain were less physically active during leisure, with stronger associations among those with higher physical effort at work. Those reporting high physical effort at work had worse (knee-related) quality of life compared to those reporting low effort at work. This highlights the importance of taking knee-loading PA at work and leisure into account when recommending exercise regimes to individuals with knee pain. With our study, we wish to contribute with new information on how knee pain impacts individuals’ overall knee-loading PA, and recommend that it be taken into consideration by health care professionals in primary care and in occupational health services.

## Electronic supplementary material

Below is the link to the electronic supplementary material.

Supplementary Material 1

## Data Availability

The datasets used and analysed during the current study are available from the corresponding author on reasonable request.

## Abbreviations

KOOS: Knee Injury and Osteoarthritis Outcome Scale
PA: Physical activity
KOA: Knee osteoarthritis
WHO: World Health Organization
HALLOA: The Halland osteoarthritis cohort
ADL: Activities in daily living
QoL: Quality of life
BMI: Body Mass Index
MVIC: The maximal voluntary isometric contraction
