# Supplementary material for: Associations between knee pain and knee-loading physical activities at work and leisure – a cross-sectional study based on accelerometer measurements
Source: BMC Musculoskelet Disord. 2025 Apr 8;26:345. doi: 10.1186/s12891-025-08589-w (PMC11980322; doi:10.1186/s12891-025-08589-w)
Supplement: Supplementary file 1 — Supplementary Material 1 [file 12891_2025_8589_MOESM1_ESM.pdf]

Additional file 1. Sensitivity analysis of table 2, including an outlier. Linear regression analysis and associations with Knee Injury Osteoarthritis Outcome Score, subscale Pain, (n=108).

		**Univariate**		**Adjusted for BMI and number of pain sites**	
	**n**	**B (95% CI)**	**p-value**	**B (95% CI)**	**p-value**
Age, (years)	108	-0.216 (-0.607; 0.175)	0.276		
Sex	108	-5.449 (-12.255;1.357)	0.115		
Body Mass Index (BMI), *(kg/m²)*	108	-0.803 (-1.502; -0.105)	**0.025**		
MVIC of quadriceps (Newton)	98	-0.006 (-0.038;0.026)	0.698		
Number of pain sites, (0-18)	106	-1.418 (-2.199; -0.638)	**<0.001**		
**Accelerometer measurements**					
Daily steps					
Total†	108	0.001 (-0.000;0.001)	0.073	0.000 (-0.000;0.001)	0.331
At work	108	0.000 (-0.001;0.001)	0.560	0.000 (-0.001;0.001)	0.963
Leisure on workdays	108	0.001 (-0.000;0.002)	0.068	0.001 (-0.000;0.002)	0.237
Upright position (minutes/day)					
Total†	108	0.041 (0.011;0.071)	**0.008**	0.032 (0.004;0.061)	**0.028**
At work	108	0.005 (-0.023;0.033)	0.713	0.002 (-0.024;0.027)	0.897
Leisure on workdays	108	0.087 (0.040;0.133)	**<0.001**	0.072 (0.027;0.116)	**0.002**
Stair walking, (minutes/day)					
Total†	108	0.166 (-0.336;0.669)	0.512	-0.006 (-0.476;0.464)	0.981
At work	108	-0.053 (-0.495;0.389)	0.814	-0.164 (-0.567;0.239)	0.422
Leisure on workdays	108	0.922 (-0.071;1.915)	0.068	0.775 (-0.128;1.697)	0.092
Sitting/lying (minutes/day)					
Total†	108	-0.029 (-0.062;0.005)	0.090	-0.022 (-0.053;0.010)	0.176
At work	108	0.002 (-0.030;0.033)	0.902	0.002 (-0.027;0.030)	0.908
Leisure on workdays	108	-0.028 (-0.066;0.009)	0.137	-0.027 (-0.061;0.007)	0.119

B = unstandardized coefficient. **KOOS =** Knee injury and Osteoarthritis Outcome Score (0-100 worst-best). **MVIC** = Maximal Voluntary Isometric Contraction. **Daily steps** = average daily steps . **Upright position =** including standing, moving, walking, running and stair walking. **Sitting/lying** = (when awake) in sitting or lying position. †Including all activities during waking hours, both working days and days off. P-value in bold typeface ≤0.005.
